# Prevalence of secondary traumatic stress in nurses: a meta-analysis of observational studies

**DOI:** 10.3389/fpubh.2026.1877091

**Published:** 2026-06-23

**Authors:** Qingyue You, Jie Li, Liyao Yang, Yinjun Zhang, Lin Chen

**Affiliations:** 1 Hemodialysis Center, Department of Nephrology, West China Hospital, Sichuan University/West China School of Nursing, Sichuan University, Chengdu, Sichuan, China; 2 Mental Health Center, West China Hospital/West China School of Nursing, Sichuan University, Chengdu, China; 3 Department of Nephrology, West China Hospital, Sichuan University/West China School of Nursing, Sichuan University, Chengdu, Sichuan, China

**Keywords:** meta-analysis, nurses, observational studies, prevalence, secondary traumatic stress

## Abstract

**Background:**

The reported prevalence of secondary traumatic stress (STS) among nurses varies considerably across studies, ranging from 22. 1 to 84.4%. This meta-analysis aimed to estimate the pooled prevalence of STS and identify potential moderating factors among nurses.

**Methods:**

From the inception of each target database to April 2026, a comprehensive search was performed across PubMed, Web of Science, Scopus, Embase, Cochrane Library, CINAHL, and PsycINFO. We calculated the pooled prevalence of STS using a random-effects model and assessed heterogeneity using the I^2^ statistic. Subgroup analyses and meta-regression were conducted to explore potential sources of heterogeneity.

**Results:**

A total of 28 studies comprising 7,090 nurses were included. The pooled prevalence of STS in nurses was 57.3% (95% CI: 49.7-64.9%). STS prevalence was significantly associated with mean age (β = −0.064, *p* = 0.023), work experience (β = −0.080, *p* = 0.040), publication year (during COVID-19: β = 0.979, *p* = 0.024; after COVID-19: β = 0.848, *p* = 0.030), and geographic region (North America: β = −0.881, *p* = 0.019; Europe: β = −1.031, *p* = 0.005).

**Conclusions:**

Our findings indicated that STS was very prevalent in nurses, and the prevalence is moderated by mean age, work experience, publication year, and geographic region. Regular STS assessments and multi-level support systems, such as early warning, peer support, and mental health training, are recommended to reduce STS risk and enhance the wellbeing of nurses.

## 1 Introduction

Nurses constitute the largest professional group in the healthcare workforce and have the closest contact with patients, undertaking critical responsibilities such as monitoring of disease progression, symptom management, psychological support, and health education (1, 2). However, clinical nursing is characterized by irregular shift schedules, high workload, sustained psychological strain, and substantial occupational risks (3). These cumulative challenges place nurses at considerable risk for occupation-related health impairments, encompassing both physical and psychological domains (4). Yet a vital but generally overlooked occupational hazard among nurses in this context is secondary traumatic stress (STS) (5).

Secondary traumatic stress is characterized by a series of physical and emotional symptoms arising from indirect exposure to traumatic events experienced by patients, including intrusion, avoidance, and hyperarousal (5). In clinical practice, nurses often witness or learn about patients' traumatic experiences, including severe injury, death, violence, and terminal suffering (6). This continuous empathic contact puts nurses at considerable risk for STS. Nurses with high STS levels tend to report mental health problems such as anxiety, depression, and post-traumatic stress disorder, as well as physical complaints like insomnia, fatigue, and somatization (7–9). Moreover, previous studies demonstrated that STS is associated with a range of adverse outcomes among nurses, including burnout, increased turnover intention, reduced job satisfaction, and diminished quality of patient care (10, 11). Therefore, it is imperative for nurse managers to prioritize the identification, prevention, and mitigation of STS among nurses.

Using a specific assessment tool, the Secondary Traumatic Stress Scale (STSS), the prevalence of STS among nurses has been examined in multiple clinical settings, such as emergency departments, intensive care units, oncology units, pediatric wards, and midwifery (12–15). However, the reported prevalence of STS varies considerably across studies, ranging from 22.1 to 84.4% (12–14, 16), with variations potentially attributable to differences in nursing specialties and demographic characteristics. For example, the reported prevalence of STS among nurses is 83.4% in emergency departments, 22.1% in intensive care units, and 84.4% in trauma care unit (12, 13, 16). In addition, variations may also be attributable to demographic characteristics, such as age and work experience, as well as geographic regions, including Asia, Europe, and North America. These variables (mean age, work experience, publication year, and geographic region) will be examined as potential moderators in this meta-analysis. To date, there has been only one meta-analysis that pooled the prevalence of STS among emergency nurses (17). However, it was confined to the emergency setting and did not synthesize prevalence estimates of STS from a broader range of observational studies across various nursing populations. Moreover, the COVID-19 pandemic has exacerbated nurses' exposure to traumatic events through recurrent patient deaths, elevated workloads due to staffing shortages, moral distress from resource allocation decisions, anxiety over insufficient personal protective equipment, and public stigma (14, 16). These compounding stressors may have elevated STS levels among nurses during and following the pandemic. This further highlights the need for an updated and comprehensive evidence synthesis. Therefore, this meta-analysis of observational studies aimed to determine the pooled prevalence of STS in nurses and identify its potential moderating factors.

## 2 Methods

Following the Preferred Reporting Items for Systematic Reviews and Meta-Analyses (PRISMA) guidelines (18), we conducted this meta-analysis of observational studies and registered the protocol in PROSPERO (CRD420261384616).

### 2.1 Search strategy

From the inception of each target database to April 2026, a systematic search was performed across PubMed, Web of Science, Scopus, Embase, Cochrane Library, CINAHL, and PsycINFO. The search strategy (Supplementary Table S1) employed Boolean logic to combine MeSH terms and related free keywords, including nurses, nursing staff, secondary traumatic stress, STS, STSS, and prevalence. Additional potential eligible studies came from manual examination of the reference lists of all included studies.

### 2.2 Eligibility criteria

Eligible studies were those that: (1) enrolled nurses as participants; (2) adopted a cross-sectional or longitudinal design; (3) diagnosed STS using the Secondary Traumatic Stress Scale with a cutoff of 38; (4) either reported the prevalence of secondary traumatic stress or contained enough information to calculate it. Excluded publications included gray literature (conference abstracts, dissertations, unpublished manuscripts), protocols, case reports, reviews, duplicate publications, and articles not written in English.

### 2.3 Literature selection and data extraction

Data were independently extracted by two reviewers, with disagreements resolved through consultation with a third reviewer. The collected information included first author, publication year, country, study design, sample size, mean age, work experience, proportion of female participants, unit category, and STS prevalence.

### 2.4 Risk of bias assessment

Using the Joanna Briggs Institute (JBI) critical appraisal checklist for prevalence studies (19), two reviewers independently rated nine domains for risk of bias. Each domain was scored as “yes” (low risk), “no” (high risk), or “unclear/not applicable.” Based on the proportion of “yes” answers, studies were classified as low (≥70%), moderate (50%−69%), or high ( ≤ 49%) risk. Any discrepancies were settled through discussion with a third reviewer to achieve consensus.

### 2.5 Statistical analysis

Statistical analyses were conducted with Stata 15.0. Given the binary nature of the prevalence outcome, a logit transformation was applied to stabilize the variance and normalize the distribution of the pooled estimates. Heterogeneity was quantified using the I^2^ statistic, with a value ≥ 50% indicating substantial heterogeneity. A DerSimonian-Laird random-effects model was then employed to calculate the pooled prevalence and its 95% confidence interval (CI) (20). Subgroup analyses by publication year, economic status, geographic region, and unit category were conducted to explore potential heterogeneity sources. In addition, univariate meta-regression was performed to assess the relationship between covariates and the pooled prevalence. Publication bias was examined using funnel plots and Egger's test, and the robustness of the findings was assessed via leave-one-out sensitivity analysis. Statistical significance was set at two-sided *P* < 0.05.

## 3 Results

### 3.1 Literature selection and characteristics

**Figure 1:**
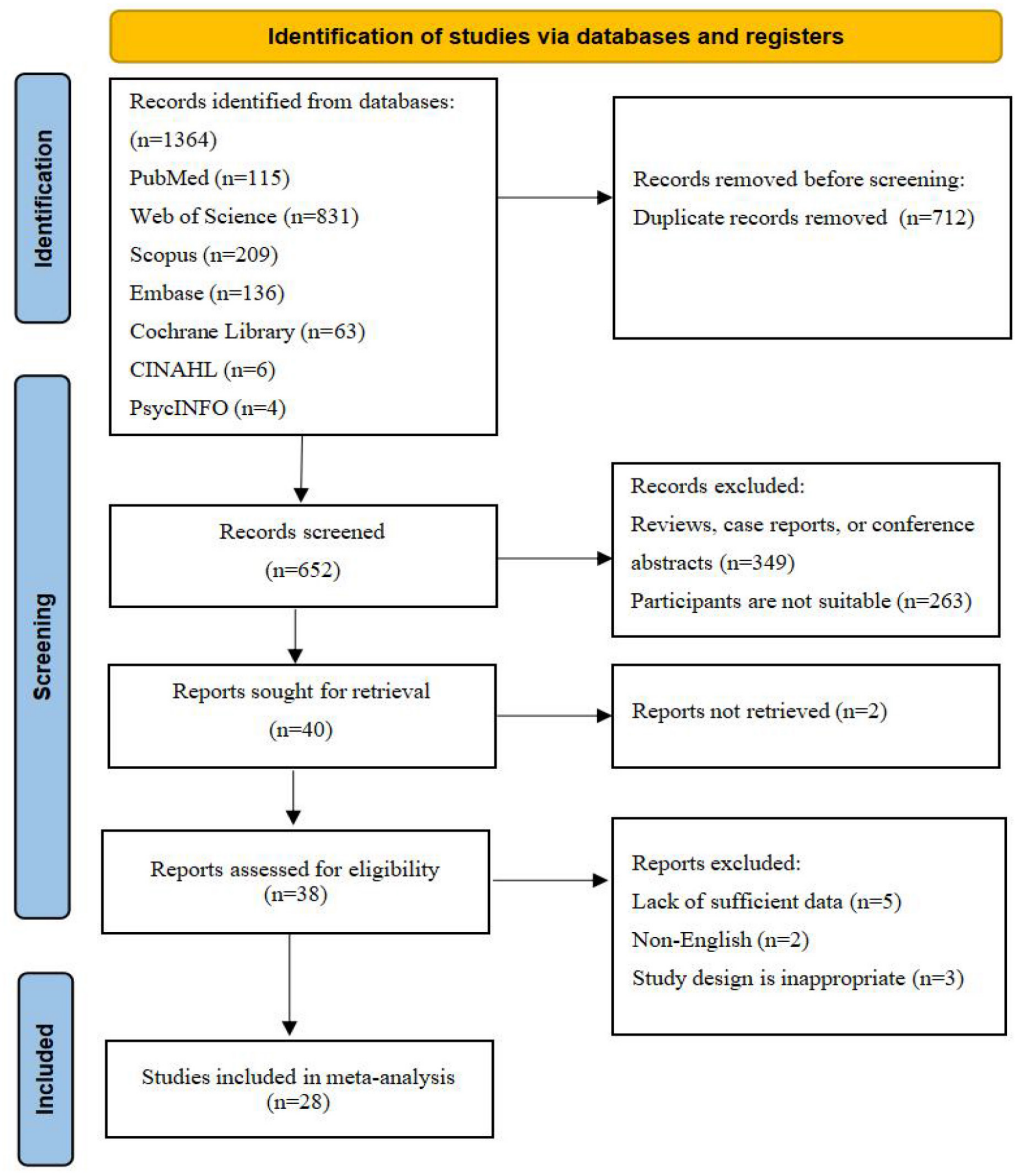
Flow diagram of study selection.

The initial database search yielded 1,364 records. After removing 712 duplicates, 652 records were screened by title and abstract. Of these, 614 records were excluded, and the remaining 38 full-text articles were assessed for eligibility. Following full-text review, 10 articles were excluded for various reasons (insufficient data, non-English publication, and inappropriate study design), leaving 28 studies that met all inclusion criteria and were included in the meta-analysis. Figure 1 summarizes the study selection process. These 28 studies involved 7,090 nurses from 14 countries. All studies employed a cross-sectional design, and most were conducted in high-income countries. Individual study sample sizes ranged from 67 to 784. Detailed characteristics of the included studies are presented in Table 1.

**Table 1:** Characteristics of included studies.

Study	Country	Study design	Sample size	Mean age (years)	Work experience (years)	Female (%)	Unit category	Prevalence (%)
Al Barmawi et al. (12)	Jordan	Cross-sectional	317	30.2	5.4	52.4	Intensive care unit	22.1
Alshammari et al. (13)	Saudi Arabia	Cross-sectional	181	29.9	7.6	50.8	Emergency department	83.4
Ariapooran et al. (14)	Iran	Cross-sectional	315	34.7	11.3	61.9	Multiple departments	51.1
Beck and Gable. (15)	USA	Cross-sectional	464	46.7	20.8	98.9	Obstetrics department	34.5
Beck et al. (32)	USA	Cross-sectional	473	50.4	16.7	99.0	Obstetrics department	23.5
Beck et al. (33)	USA	Cross-sectional	175	49.8	22.2	97.1	Intensive care unit	49.1
Cai et al. (34)	China	Cross-sectional	391	34.9	NA	97.4	Oncology department	68.3
Civljak et al. (35)	Croatia	Cross-sectional	158	35.3	NA	94.7	Multiple departments	48.1
Comparcini et al. (10)	Italy	Cross-sectional	271	39	14.7	71.2	Multiple departments	28.4
Dominguez-Gomez and Rutledge. (21)	USA	Cross-sectional	67	42.6	14	77.6	Emergency department	32.8
Duffy et al. (36)	Ireland	Cross-sectional	105	40	19	95.2	Emergency department	63.8
Erkin et al. (37)	Turkey	Cross-sectional	205	NA	NA	76.1	Multiple departments	80.5
Frazier. (38)	USA	Cross-sectional	169	36	NA	76.9	Emergency department	79.3
Haji Ali Begloo et al. (28)	Iran	Cross-sectional	123	32.2	8.6	NA	Intensive care unit	69.1
He et al. (39)	China	Cross-sectional	784	NA	NA	98.7	Multiple departments	63.8
Hu et al. (40)	China	Cross-sectional	229	28.3	8.6	86.5	Intensive care unit	63.3
Kellogg et al. (41)	USA	Cross-sectional	334	41.3	16	98.2	Pediatric department	50.9
Lim et al. (42)	South Korea	Cross-sectional	147	29.8	5.4	98.6	Intensive care unit	68.0
Lubbad et al. (43)	Palestine	Cross-sectional	293	NA	NA	54.9	Oncology department	62.6
Morrison and Joy. (44)	UK	Cross-sectional	80	40	10	77.5	Emergency department	38.8
Nicholls et al. (45)	USA	Cross-sectional	144	37.3	12.6	99.3	Obstetrics department	35.4
Ratrout and Hamdan-Mansour. (46)	Jordan	Cross-sectional	202	27.9	5.4	38.6	Emergency department	74.8
Salameh et al. (47)	Palestine	Cross-sectional	189	NA	NA	34.9	Emergency department	77.2
Scott et al. (48)	UK	Cross-sectional	246	NA	NA	91.9	Intensive care unit	39.8
Tsouvelas et al. (49)	Greece	Cross-sectional	222	42.3	NA	87.4	Multiple departments	65.8
Woo and Kim. (16)	South Korea	Cross-sectional	186	NA	1.9	88.7	Trauma care unit	84.4
Yao et al. (50)	China	Cross-sectional	434	NA	NA	91.9	Multiple departments	66.1
Yehene et al. (51)	Israel	Cross-sectional	186	36.5	10.1	84.4	Pediatric department	80.0

NA, Not available.

### 3.2 Risk of bias assessment results

According to the Joanna Briggs Institute (JBI) critical appraisal checklist, 11 studies (39.3%) were assessed as having a low risk of bias, whereas the remaining 17 (60.7%) had a moderate risk of bias. In terms of the individual domains, the sample frame was appropriate in 27 studies (96.4%), the sampling method had high bias risk in all 28 (100%) due to convenience sampling, sample size was adequate in only 9 (32.1%), and unclear in 19 (67.9%). All 28 studies satisfied in description, data coverage, valid methods, reliable measurement, and appropriate analysis. For response rate, 11 (39.3%) were adequate, 4 (41.3%) inadequate, and 13 (46.4%) unclear.

### 3.3 Pooled prevalence of STS in nurses

**Figure 2:**
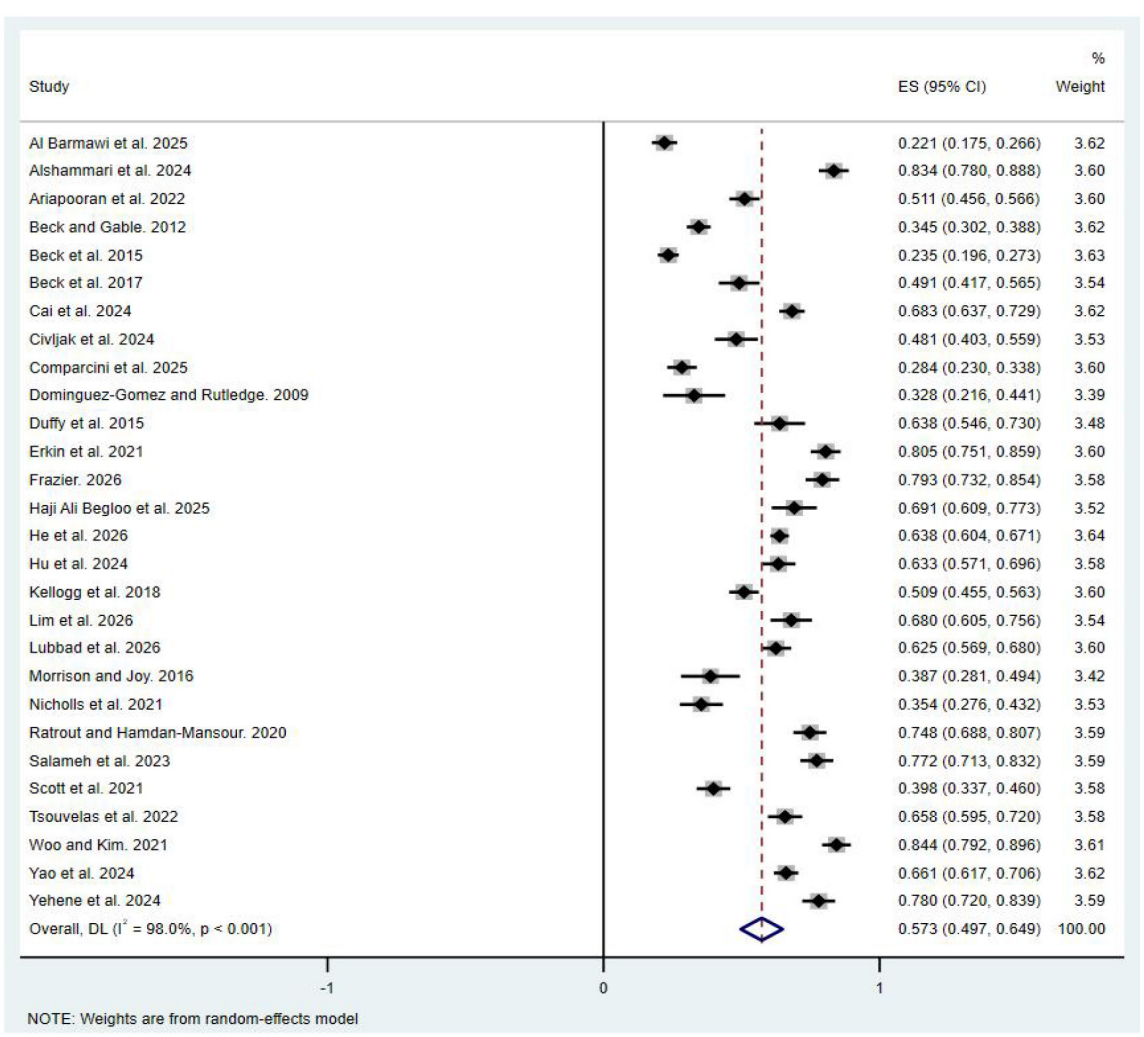
Forest plot of pooled prevalence of secondary traumatic stress in nurses.

A substantial heterogeneity (I^2^ = 98.0%) was observed across studies. Therefore, we employed a random-effects model, which yielded a pooled STS prevalence of 57.3% (95% CI: 49.7–64.9%) in nurses (Figure 2).

### 3.4 Subgroup analysis and meta-regression analysis

The results of the subgroup analyses are summarized in Table 2. The pooled prevalence of STS in nurses varied significantly by publication year (Supplementary Figure S1), with the lowest prevalence observed before the COVID-19 outbreak (41.8%, 95% CI: 31.2–52.3%) and higher estimates during (63.7%, 95% CI: 51.1**–**76.4%) and after (61.5%, 95% CI: 51.1–71.9%) the outbreak. By economic status, no significant difference (Supplementary Figure S2) was found between high-income (54.8%, 95% CI: 43.9–65.6%) and middle-income countries (61.8%, 95% CI: 51.5–72.0%). When analyzed by geographic region (Supplementary Figure S3), the pooled prevalence was highest in Asia (67.5%, 95% CI: 59.0–76.0%), followed by Europe (47.4%, 95% CI: 34.4–60.4%) and North America (43.7%, 95% CI: 29.3–58.1%). Subgroup analysis by unit category (Supplementary Figure S4) revealed no significant difference (*P* = 0.599) among acute and critical care departments (60.6%, 95% CI: 48.6–72.5%), mixed departments (57.7%, 95% CI: 46.0–69.5%), and specialty wards (50.4%, 95% CI: 34.7–66.1%).

**Table 2:** Subgroup analyses of the pooled prevalence of secondary traumatic stress in nurses.

Subgroups	Number of studies	Prevalence (95% CI)	Heterogeneity		*P* values across subgroups
			I^2^	*P* values	
Publication year					
Before COVID-19 outbreak	7	41.8% (31.2–52.3%)	95.0%	*P* < 0.001	0.009
During COVID-19 outbreak	8	63.7% (51.1–76.4%)	97.2%	*P* < 0.001	
After COVID-19 outbreak	13	61.5% (51.1–71.9%)	98.0%	*P* < 0.001	
Economic status					
High income	17	54.8% (43.9–65.6%)	98.3%	*P* < 0.001	0.357
Middle income	11	61.8% (51.5–72.0%)	97.5%	*P* < 0.001	
Geographic region					
Asia	16	67.5% (59.0–76.0%)	97.4%	*P* < 0.001	0.004
North America	6	43.7% (29.3–58.1%)	97.7%	*P* < 0.001	
Europe	6	47.4% (34.4–60.4%)	95.0%	*P* < 0.001	
Unit category					
Acute and critical care departments	15	60.6% (48.6–72.5%)	97.9%	*P* < 0.001	0.599
Mixed departments	7	57.7% (46.0–69.5%)	97.3%	*P* < 0.001	
Specialty wards	6	50.4% (34.7–66.1%)	98.5%	*P* < 0.001	

Univariate meta-regression analysis (Table 3) demonstrated that mean age, work experience, publication year, and geographic regionwere significantly associated with the pooled prevalence of STS in nurses (*P* < 0.05). In contrast, none of the other moderators examined, including sample size, proportion of female participants, economic status, and unit category, were significantly associated (all *P* > 0.05).

**Table 3:** The results of meta-regression.

Variables	Number of studies	Coefficient	Standard error	95% CI	*t* values	*P* values
Sample size	28	−0.0009	0.0011	(−0.0032, 0.0013)	−0.88	0.388
Mean age	21	−0.0636	0.0258	(−0.1176, −0.0097)	−2.47	0.023
Work experience	18	−0.0797	0.0356	(−0.1552, −0.0043)	−2.24	0.040
Proportion of female participants	28	−0.0099	0.0086	(−0.0276, 0.0078)	−1.15	0.260
Publication year (ref: before COVID-19 outbreak)						
During COVID-19 outbreak	8	0.9790	0.4085	(0.1378, 1.8203)	2.40	0.024
After COVID-19 outbreak	13	0.8484	0.3689	(0.0867, 1.6101)	2.29	0.030
Economic status (ref: high income)						
Middle income	11	0.2591	0.3398	(−0.4394, 0.9576)	0.76	0.453
Geographic region (ref: Asia)						
North America	6	−0.8810	0.3526	(−0.3397, −0.2385)	−2.50	0.019
Europe	6	−1.0306	0.3336	(−1.7177, −0.3435)	−3.09	0.005
Unit category (ref: acute and critical care departments)						
Mixed departments	7	−0.1476	0.4008	(−0.9731, 0.6780)	−0.37	0.716
Specialty wards	6	−0.4632	0.4007	(−1.2886, 0.3622)	−1.16	0.259

### 3.5 Publication bias and sensitivity analysis

**Figure 3:**
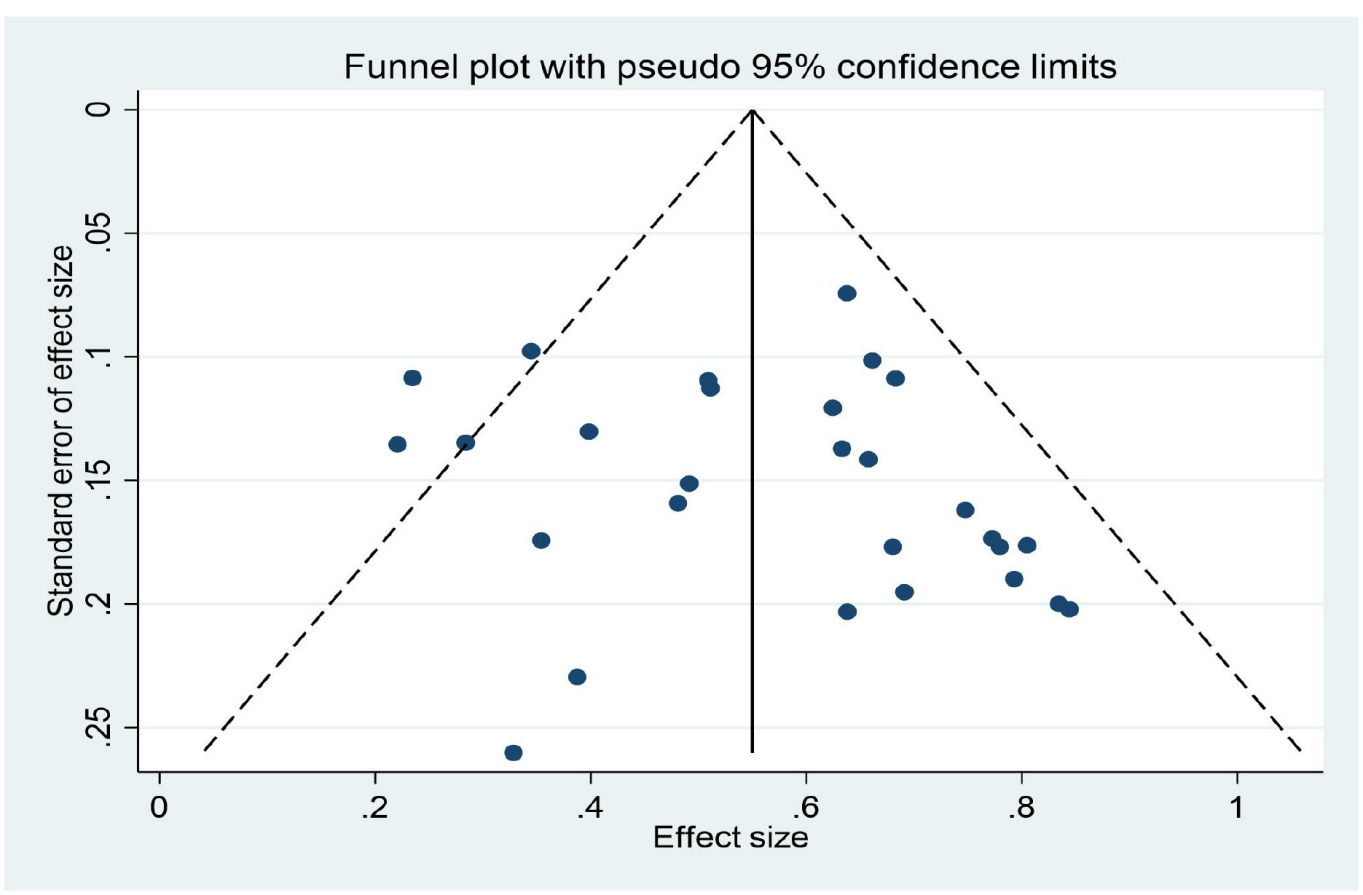
Funnel plot.

The funnel plot exhibited some asymmetry (Figure 3), suggesting possible publication bias or other sources of heterogeneity. However, Egger's test (Supplementary Figure S5) was not statistically significant (*P* > 0.05), indicating no significant evidence of funnel plot asymmetry, and sensitivity analysis confirmed the robustness of the findings (Supplementary Table S3).

## 4 Discussion

To our knowledge, this is the first meta-analysis of observational studies to determine the prevalence of STS in nurses. The pooled analysis of the 28 included studies showed that the overall prevalence of STS among the 7,090 nurses was 57.3%. However, given the extremely high heterogeneity across studies, this estimate should be interpreted as an approximate summary across highly diverse nursing contexts, including differences in geographic regions, clinical settings, pandemic periods, and nurse demographics, rather than as a stable or universal prevalence applicable to all nurses. This finding is significantly lower than the prevalence reported in a previous meta-analysis estimating STS among emergency nurses (17). The primary etiology of STS lies in direct or indirect exposure to traumatic events (12). Emergency nurses, who endure chronic high stress and heavy emotional loads, are more frequently exposed to life-threatening emergencies, patient deaths, distressed relatives, and workplace violence than nurses in general clinical settings, thereby significantly contributing to a substantially increased risk of STS (13, 21). In this study, a broader range of nursing populations (emergency department, intensive care unit, pediatrics, and oncology nurses) was encompassed across the 28 included observational studies, thereby yielding a lower pooled prevalence of STS. However, the pooled STS prevalence of 57.3% still suggested that STS is a common occupational psychological distress in nurses, necessitating considerable attention from nursing administrators. Therefore, regular screening for STS should be implemented among nurses, who should also be encouraged to voluntarily report their emotional condition. Moreover, targeted interventions, such as cognitive behavioral therapy, mindfulness-based stress reduction, peer support programs, and resilience training, should be provided to alleviate their STS symptoms (22).

In subgroup analysis and meta-regression, publication year and geographic region were observed to be associated with the pooled prevalence of STS in nurses. Specifically, the prevalence of STS among nurses was highest during the COVID-19 pandemic outbreak, which is consistent with the findings of previous studies. The COVID-19 pandemic was a global public health crisis that pushed healthcare systems in various countries to the brink of collapse (23). During the pandemic, nurses were daily exposed to a large number of critically ill patients. Infection risk, discomfort caused by protective equipment, and prolonged shifts led to physical and mental exhaustion (13). Meanwhile, internal psychological support and peer debriefing were reduced due to staffing shortages, and home-based social isolation cut off external emotional support (24). Moreover, the acute shortage of medical resources forced nurses to frequently confront ethical dilemmas of selective treatment, an experience that in itself constituted a severe psychological trauma (14). It is noteworthy that the pooled prevalence of STS among nurses in North America and Europe was found to be lower than that in Asia. Previous studies have suggested that in some Asian settings, low compensation, poor recognition, and unclear career progression may contribute to a substantial brain drain of skilled nursing professionals overseas (25). Consequently, many Asian countries face a critical nurse shortage, which may force each nurse to bear a workload multiple times above the normal level, potentially rendering them more vulnerable to stress responses (26). Additionally, seeking psychological help remains stigmatized in certain Asian cultures, where it may be construed as a sign of frailty, leading nurses to hide their psychological distress to avoid being labeled as vulnerable (27). These hypotheses, drawn from external literature, suggest an urgent need for future research to examine the roles of culturally tailored anti-stigma interventions, improved staffing and resources, and robust institutional psychological support, particularly in Asian countries and in preparation for future health crises.

Meta-regression indicated that mean age and work experience were significant moderators of STS among nurses. Specifically, studies with older mean nurse age and longer mean work experience reported lower STS prevalence. These findings are consistent with those of many previous studies (10, 28). First, at the individual level, it has been proposed that older and more experienced nurses may gradually adapt to high-stress environments over long-term work, accumulating many psychological skills for coping with traumatic events such as emotional isolation and cognitive restructuring (22). They may more effectively regulate the negative emotions brought by empathy and are less likely to be overwhelmed by the pain of patients. Furthermore, experienced nurses tend to have a clearer understanding of their professional role and believe in their ability to help patients. This sense of accomplishment may buffer against STS, whereas new nurses might feel powerless due to the discrepancy between idealistic expectations and reality (22). Finally, older nurses generally have more stable collegial relationships and a stronger voice within the organization, which makes it easier for them to access psychological support from external sources, thereby reducing the risk of STS (22). However, given that the meta-regression findings are based on study-level aggregate data rather than individual nurse-level data, these associations should be interpreted as ecological-level patterns rather than evidence of individual-level causal effects. Therefore, drawing on the broader literature (29–31), targeted interventions such as coping skills training, role clarity enhancement, and access to external psychological support may be prioritized for younger and less experienced nurses to reduce their STS, although these recommendations are not direct findings of the present prevalence meta-analysis.

## 5 Limitations

Several limitations should be considered. First, although no significant publication bias was detected, the restriction to English-language articles and omission of gray literature could have introduced such bias. Second, because all included studies were conducted in high-income and middle-income countries, our findings may not reflect the conditions specific to low-income countries. Third, all included studies employed cross-sectional designs, which preclude causal inferences regarding the identified moderators. Fourth, all included studies employed convenience sampling, which may either overestimate or underestimate the true STS prevalence depending on whether highly distressed nurses were more or less likely to participate. This potential bias limits the representativeness of the estimate. Fifth, consistent with other prevalence meta-analyses, the included studies exhibited substantial heterogeneity, which was only partly explained by mean age, work experience, publication year, and geographic region. Unmeasured factors likely contributed to the observed variance, including differences in clinical contexts, organizational support systems, trauma training, cultural attitudes toward emotional distress, and STS measurement instruments. Finally, the meta-regression analyses were univariate and based on study-level aggregate data, and some moderator analyses included a limited number of studies, which may have reduced statistical power, and unmeasured confounders could not be accounted for in the univariate model. Therefore, the observed associations should be interpreted as ecological-level patterns rather than individual-level causal effects.

## 6 Conclusion

Our findings demonstrated that more than half of nurses are impacted by STS, and the prevalence is moderated by mean age, work experience, publication year, and geographic region. Given the substantial heterogeneity across studies, these estimates should be interpreted as reflecting a context-dependent burden rather than a universal figure. Therefore, healthcare administrators should implement regular STS evaluations for nurses and create multi-level support systems that comprise early warning mechanisms, peer support programs, and periodic mental health training, so as to reduce STS risk and alleviate its impact on the wellbeing of nurses.

## Data Availability

The original contributions presented in the study are included in the article/Supplementary material, further inquiries can be directed to the corresponding author.

## References

[1] Zhou XH Duan DF Chen L Zhang YJ Gong S Chen Q. Relationship between professional quality of life and career success in nurses: a latent profile analysis. Int Nurs Rev. (2025) 72:e70044. doi: 10.1111/inr.70044 40528567

[2] Kim Y Yu M. Hospital nurses' professional quality of life model: a cross-sectional study based on the expanded job demands-resources model. J Nurs Manag. (2025) 2025:7500360. doi: 10.1155/jonm/7500360 40230448 PMC11996276

[3] Butler S. Understanding burnout in nurses: identification and coping strategies. Br J Nurs. (2025) 34:220–4. doi: 10.12968/bjon.2024.0244 39969835

[4] Badawy W Shalaby SA Shaban M. Burnout and compassion fatigue among ICU nurses: a systematic review of predictors, protective factors and workforce retention interventions. Nurs Crit Care. (2026) 31:e70377. doi: 10.1111/nicc.70377 41725578

[5] Kellogg MB. Secondary traumatic stress in nursing: a walker and avant concept analysis. ANS Adv Nurs Sci. (2021) 44:157–70. doi: 10.1097/ANS.0000000000000338 33394584

[6] Lee MS Shin S Hong E. Factors affecting secondary traumatic stress of nurses caring for COVID-19 patients in South Korea. Int J Environ Res Public Health. (2021) 18:6843. doi: 10.3390/ijerph18136843 34202283 PMC8297365

[7] Zhang J Wang X Chen O Zhang J. Professional quality of life influences sleep and well-being in nurses: a cross-sectional study. Appl Nurs Res. (2025) 84:151980. doi: 10.1016/j.apnr.2025.151980 40744550

[8] Tofthagen C Chesak S Mielke C Lindroth H Foster R Lubrano B. Relationships between post-traumatic stress, self-compassion, sleep, anxiety, depressive symptoms, and intensive care unit nurses' intent to leave their jobs. J Nurs Adm. (2026) 56:92–6. doi: 10.1097/NNA.0000000000001686 41568837

[9] Park H Kim H Kim H. A scoping review of secondary traumatic stress in nurses working in the emergency department or trauma care settings. J Adv Nurs. (2025) 81:5304–14. doi: 10.1111/jan.16938 40249754 PMC12371812

[10] Comparcini D Simonetti V Totaro M Apicella A Galli F Toccaceli A . Impact of traumatic stress on nurses' work ability, job satisfaction, turnover and intention to leave: a cross-sectional study. J Adv Nurs. (2025) 81:6504–14. doi: 10.1111/jan.16796 39930285

[11] Mohamed Z Forawi S. Unit-based differences in compassion satisfaction, burnout, secondary traumatic stress, and turnover intention among nurses in a tertiary hospital in Abu Dhabi. Front Public Health. (2025) 13:1686060. doi: 10.3389/fpubh.2025.1686060 41235204 PMC12605496

[12] Al Barmawi M Shahrouri BE Al Hadid L Alzoubi MM Al-Mugheed K Alabdullah AAS . Measuring the prevalence, warning signs, and preventive measures of secondary traumatic stress among critical care nurses. BMC Psychiatry. (2025) 25:450. doi: 10.1186/s12888-025-06840-1 40325395 PMC12054241

[13] Alshammari B Alanazi NF Kreedi F Alshammari F Alkubati SA Alrasheeday A . Exposure to secondary traumatic stress and its related factors among emergency nurses in Saudi Arabia: a mixed method study. BMC Nurs. (2024) 23:337. doi: 10.1186/s12912-024-02018-4 38762742 PMC11102619

[14] Ariapooran S Ahadi B Khezeli M. Depression, anxiety, and suicidal ideation in nurses with and without symptoms of secondary traumatic stress during the COVID-19 outbreak. Arch Psychiatr Nurs. (2022) 37:76–81. doi: 10.1016/j.apnu.2021.05.005 35337442 PMC8938317

[15] Beck CT Gable RK. A mixed methods study of secondary traumatic stress in labor and delivery nurses. J Obstet Gynecol Neonatal Nurs. (2012) 41:747–60. doi: 10.1111/j.1552-6909.2012.01386.x 22788967

[16] Woo MJ Kim DH. Factors associated with secondary traumatic stress among nurses in regional trauma centers in South Korea: a descriptive correlational study. J Emerg Nurs. (2021) 47:400–11. doi: 10.1016/j.jen.2020.08.006 33229000

[17] Xu Z Zhao B Zhang Z Wang X Jiang Y Zhang M . Prevalence and associated factors of secondary traumatic stress in emergency nurses: a systematic review and meta-analysis. Eur J Psychotraumatol. (2024) 15:2321761. doi: 10.1080/20008066.2024.2321761 38426665 PMC10911249

[18] Page MJ McKenzie JE Bossuyt PM Boutron I Hoffmann TC Mulrow CD . The PRISMA 2020 statement: an updated guideline for reporting systematic reviews. BMJ. (2021) 372:n71. doi: 10.1136/bmj.n71 33782057 PMC8005924

[19] Munn Z Moola S Lisy K Riitano D Tufanaru C. Methodological guidance for systematic reviews of observational epidemiological studies reporting prevalence and cumulative incidence data. Int J Evid Based Healthc. (2015) 13:147–53. doi: 10.1097/XEB.0000000000000054 26317388

[20] Higgins JP Thompson SG Deeks JJ Altman DG. Measuring inconsistency in meta-analyses. BMJ. (2003) 327:557–60. doi: 10.1136/bmj.327.7414.557 12958120 PMC192859

[21] Dominguez-Gomez E Rutledge DN. Prevalence of secondary traumatic stress among emergency nurses. J Emerg Nurs. (2009) 35:199–274. doi: 10.1016/j.jen.2008.05.003 19446123

[22] Zhang H Xia Z Yu S Shi H Meng Y Dator WL. Interventions for compassion fatigue, burnout, and secondary traumatic stress in nurses: a systematic review and network meta-analysis. Nurs Health Sci. (2025) 27:e70042. doi: 10.1111/nhs.70042 39887608

[23] Sung HK Lee KS Lee J Oh MH Kim JY Choi YK . Delayed at the door: impact of pandemic response policies on emergency and critical care in Korea: an interrupted time-series analysis. J Korean Med Sci. (2026) 41:e64. doi: 10.3346/jkms.2026.41.e64 41775276 PMC12956905

[24] Mazibu D Downing C Rasesemola R. Experiences of critical care nurses infected with COVID-19 in a Saudi academic hospital. Curationis. (2025) 48:e1–9. doi: 10.4102/curationis.v48i1.2735 40336382 PMC12135194

[25] Abou Hashish EA Alnajjar HA. Brain drain and retention strategies: lived experience of expatriate nurses in Saudi Arabia: challenges and implications. J Nurs Manag. (2025) 2025:9947313. doi: 10.1155/jonm/9947313 40808861 PMC12349981

[26] Venjulien Sangalang Japon R. From burnout to bottlenecks: the multidimensional impact of nurse shortages in hospital operations and patient outcomes. Int J Res Innov Appl Sci. (2025) 10:676–81. doi: 10.51584/IJRIAS.2025.100800059

[27] Tan J Shi W Yuan GF Lowe SR Liu J. Perceived barriers and influencing factors of psychological help-seeking amongst Chinese nurses exposed to COVID-19. J Res Nurs. (2024) 29:203–13. doi: 10.1177/17449871241236387 38883255 PMC11179599

[28] Haji Ali Begloo R Oujian P Nourian M Wilson M Varzeshnejad M. Assessment of secondary traumatic stress symptoms and related factors among NICU nurses. Perspect Psychiatr Care. (2025) 2025:1153950. doi: 10.1155/ppc/1153950

[29] Chen YH Chang YY Wang IT Chang SH. When caring hurts: the buffering role of social support in emergency department nurses' coping with secondary traumatic stress. J Nurs Manag. (2026) 2026:e8247254. doi: 10.1155/jonm/8247254 42189104 PMC13205575

[30] Bommen S Nicholls H Billings J. ‘Helper' or ‘punisher'? A qualitative study exploring staff experiences of treating severe and complex eating disorder presentations in inpatient settings. J Eat Disord. (2023) 11:216. doi: 10.1186/s40337-023-00938-1 38062517 PMC10704651

[31] Carne B Furyk J. Supporting clinicians post exposure to potentially traumatic events: emergency department peer support program evaluation. Emerg Med Australas. (2025) 37:e14518. doi: 10.1111/1742-6723.14518 39397272 PMC11744404

[32] Beck CT LoGiudice J Gable RK. A mixed-methods study of secondary traumatic stress in certified nurse-midwives: shaken belief in the birth process. J Midwifery Womens Health. (2015) 60:16–23. doi: 10.1111/jmwh.12221 25644069

[33] Beck CT Cusson RM Gable RK. Secondary traumatic stress in NICU nurses: a mixed-methods study. Adv Neonatal Care. (2017) 17:478–88. doi: 10.1097/ANC.0000000000000428 28914626

[34] Cai Y Liu M Li Y Li J Geng J Liu X . Secondary traumatic stress and vicarious posttraumatic growth in oncology nurses: the mediating role of empathy. Front Public Health. (2024) 12:1454998. doi: 10.3389/fpubh.2024.1454998 39354994 PMC11442218

[35] Civljak M Stivic I Puljak L. Secondary traumatic stress in working nurses studying part time in a bachelor or master's nursing program in Croatia: a cross-sectional study. BMC Nurs. (2024) 23:22. doi: 10.1186/s12912-023-01691-1 38183032 PMC10768158

[36] Duffy E Avalos G Dowling M. Secondary traumatic stress among emergency nurses: a cross-sectional study. Int Emerg Nurs. (2015) 23:53–8. doi: 10.1016/j.ienj.2014.05.001 24927978

[37] Erkin Ö Konakçi G Duran S. Secondary traumatic stress in nurses working with patients with suspected/confirmed COVID-19 in Turkey. Perspect Psychiatr Care. (2021) 57:1664–72. doi: 10.1111/ppc.12733 33522626

[38] Frazier M. Secondary traumatic stress in emergency room nurses: a cross-sectional study. Int Emerg Nurs. (2026) 85:101767. doi: 10.1016/j.ienj.2026.101767 41671713

[39] He Y Yun B Liao X Xie G Li Y Zhang J . Exploring the mediating role of rumination on empathy and secondary traumatic stress in nurses: a cross-sectional investigation. BMC Nurs. (2026) 25:72. doi: 10.1186/s12912-025-04285-1 41491685 PMC12829024

[40] Hu M Zhang H Wu C Li L Liang X Zhang Y . Relationship between moral resilience and secondary traumatic stress among ICU nurses: a cross-sectional study. Nurs Crit Care. (2024) 29:1363–72. doi: 10.1111/nicc.13120 39072948

[41] Kellogg MB Knight M Dowling JS Crawford SL. Secondary traumatic stress in pediatric nurses. J Pediatr Nurs. (2018) 43:97–103. doi: 10.1016/j.pedn.2018.08.016 30473163

[42] Lim H Lee H Kim S Kim H Park C Lee Y. Experiences and influencing factors of secondary traumatic stress in neonatal intensive care unit nurses: a mixed-methods study. J Pediatr Nurs. (2026) 87:443–50. doi: 10.1016/j.pedn.2026.01.046 41653763

[43] Lubbad A Tlili MA Amro N Missaoui N. Predictors of secondary traumatic stress among oncology nurses in Palestine. Asian Pac J Cancer Prev. (2026) 27:705–13. doi: 10.31557/APJCP.2026.27.2.705 41660929 PMC13499579

[44] Morrison LE Joy JP. Secondary traumatic stress in the emergency department. J Adv Nurs. (2016) 72:2894–906. doi: 10.1111/jan.13030 27221701

[45] Nicholls EM Hermann RM Giordano NA Trotta RL. Secondary traumatic stress among labor and delivery nurses. MCN Am J Matern Child Nurs. (2021) 46:14–20. doi: 10.1097/NMC.0000000000000674 33284241

[46] Ratrout HF Hamdan-Mansour AM. Secondary traumatic stress among emergency nurses: prevalence, predictors, and consequences. Int J Nurs Pract. (2020) 26:e12767. doi: 10.1111/ijn.12767 31328356

[47] Salameh B Daibes AG Qaddumi J. Assessing the prevalence, predictors, and consequences of secondary traumatic stress among emergency nurses in Palestine during the COVID-19 pandemic. SAGE Open Nurs. (2023) 9:23779608231207224. doi: 10.1177/23779608231207224 37830081 PMC10566272

[48] Scott Z O'Curry S Mastroyannopoulou K. Factors associated with secondary traumatic stress and burnout in neonatal care staff: a cross-sectional survey study. Infant Ment Health J. (2021) 42:299–309. doi: 10.1002/imhj.21907 33449411

[49] Tsouvelas G Kalaitzaki A Tamiolaki A Rovithis M Konstantakopoulos G. Secondary traumatic stress and dissociative coping strategies in nurses during the COVID-19 pandemic: the protective role of resilience. Arch Psychiatr Nurs. (2022) 41:264–70. doi: 10.1016/j.apnu.2022.08.010 36428058 PMC9428110

[50] Yao J Zhou X Xu D Liu T Gui Y Huang Y. Current status and influencing factors of secondary traumatic stress in emergency and intensive care nurses: a cross-sectional analysis. Psychol Res Behav Manag. (2024) 17:567–76. doi: 10.2147/PRBM.S444205 38379635 PMC10876876

[51] Yehene E Asherman A Goldzweig G Simana H Brezner A. Secondary traumatic stress among pediatric nurses: relationship to peer-organizational support and emotional labor strategies. J Pediatr Nurs. (2024) 74:92–100. doi: 10.1016/j.pedn.2023.11.019 38029691

