# Supplementary material for: Prevalence of secondary traumatic stress in nurses: a meta-analysis of observational studies
Source: Front Public Health. 2026 Jun 23;14:1877091. doi: 10.3389/fpubh.2026.1877091 (PMC13337893; doi:10.3389/fpubh.2026.1877091)
Supplement: Supplementary file 1 [file Data_Sheet_1.docx]

**Supplemental Materials**

Table S1. The search strategies.

| Databases | Step | Search Strategies |
| --- | --- | --- |
| PubMed | #1 | "nurses"[Mesh Terms] Sort by: Best match |
|  | #2 | "nursing staff"[Mesh Terms] Sort by: Best match |
|  | #3 | "nurses"[Title/Abstract] OR "nursing staff"[Title/Abstract] OR "registered nurses"[Title/Abstract] OR "clinical nurses"[Title/Abstract] Sort by: Best match |
|  | #4 | "secondary traumatic stress"[Title/Abstract] OR "STS"[Title/Abstract] OR "secondary trauma"[Title/Abstract] OR "Secondary Traumatic Stress Scale"[Title/Abstract] OR "STSS"[Title/Abstract] Sort by: Best match |
|  | #5 | "prevalence"[Mesh Terms] Sort by: Best match |
|  | #6 | "e[pidemiology](https://www.ncbi.nlm.nih.gov/mesh/68004813)"[Mesh Terms] Sort by: Best match |
|  | #7 | "prevalence"[Title/Abstract] OR "incidence"[Title/Abstract] OR "proportion"[Title/Abstract] OR "e[pidemiology](https://www.ncbi.nlm.nih.gov/mesh/68004813)"[Title/Abstract] Sort by: Best match |
|  | #8 | #1 OR #2 OR #3 Sort by: Best match |
|  | #9 | #5 OR #6 OR #7 Sort by: Best match |
|  | #10 | #4 AND #8 AND #9 Sort by: Best match |
| Web of Science | #1 | TS=([nurses](https://www.ncbi.nlm.nih.gov/mesh/68009369)) OR TS=(nursing staff) OR TS=(registered nurses) OR TS=(clinical nurses) |
|  | #2 | **Ts=(**secondary traumatic stress**) OR Ts=(STS) OR Ts=(**secondary trauma**) OR** TS=(Secondary Traumatic Stress Scale) **OR Ts=(STSS)** |
|  | #3 | TS=(prevalence) OR **TS=(incidence) OR TS=(proportion**) **OR TS=(**e[pidemiology](https://www.ncbi.nlm.nih.gov/mesh/68004813)) |
|  | #4 | #1 AND #2 AND #3 |
| Scopus | #1 | ABS("[nurses](https://www.ncbi.nlm.nih.gov/mesh/68009369)" OR "nursing staff" OR "registered nurses" OR "clinical nurses") |
|  | #2 | ABS("secondary traumatic stress" OR "STS" OR "secondary trauma" OR "Secondary Traumatic Stress Scale" OR "STSS") |
|  | #3 | ABS ("prevalence" OR "incidence" OR "proportion" OR "e[pidemiology](https://www.ncbi.nlm.nih.gov/mesh/68004813)") |
|  | #4 | #1 AND #2 AND #3 |
| Embase | **#1** | '[nurses](https://www.ncbi.nlm.nih.gov/mesh/68009369)'/exp |
|  | **#2** | 'nursing staff'/exp |
|  | **#3** | 'nurses':ti,ab,kw OR 'nursing staff':ti,ab,kw OR 'registered nurses':ti,ab,kw OR 'clinical nurses':ti,ab,kw |
|  | **#4** | 'secondary traumatic stress'/exp |
|  | **#5** | 'secondary traumatic stress'**:ti,ab,kw OR** 'STS'**:ti,ab,kw OR** 'secondary trauma'**:ti,ab,kw OR** 'Secondary Traumatic Stress Scale'**:ti,ab,kw OR** 'STSS'**:ti,ab,kw** |
|  | **#6** | 'prevalence**':ti,ab,kw OR 'incidence':ti,ab,kw OR 'proportion':ti,ab,kw OR '**e[pidemiology](https://www.ncbi.nlm.nih.gov/mesh/68004813)**':ti,ab,kw** |
|  | **#7** | #1 OR #2 OR #3 |
|  | **#8** | #4 OR #5 |
|  | **#9** | #6 AND #7 AND #8 |
| Cochrane Library | #1 | **MeSH descriptor: [Nursing staff] explode all trees** |
|  | #2 | (nurses):ti,ab,kw OR (nursing staff):ti,ab,kw OR (registered nurses):ti,ab,kw OR (clinical nurses):ti,ab,kw |
|  | #3 | **(**secondary traumatic stress**):ti,ab,kw OR (STS):ti,ab,kw OR (**secondary trauma**):ti,ab,kw OR (**Secondary Traumatic Stress Scale**):ti,ab,kw OR (STSS):ti,ab,kw** |
|  | #4 | (prevalence)**:ti,ab,kw OR (incidence**)**:ti,ab,kw OR (proportion**)**:ti,ab,kw OR (**e[pidemiology](https://www.ncbi.nlm.nih.gov/mesh/68004813))**:ti,ab,kw** |
|  | #5 | #1 OR #2 |
|  | #6 | #3 AND #4 AND #5 |
| CINAHL | #1 | TI [nurses](https://www.ncbi.nlm.nih.gov/mesh/68009369) OR TI nursing staff OR TI registered nurses OR TI clinical nurses |
|  | #2 | **TI** secondary traumatic stress **OR TI STS OR TI** secondary trauma **OR** TI Secondary Traumatic Stress Scale **OR TI STSS** |
|  | #3 | TI prevalence OR **TI incidence** OR **TI proportion OR TI** e[pidemiology](https://www.ncbi.nlm.nih.gov/mesh/68004813) |
|  | #4 | #1 AND #2 AND #3 |
| PsycINFO | #1 | TI [nurses](https://www.ncbi.nlm.nih.gov/mesh/68009369) OR TI nursing staff OR TI registered nurses OR TI clinical nurses |
|  | #2 | **TI** secondary traumatic stress **OR TI STS OR TI** secondary trauma **OR** TI Secondary Traumatic Stress Scale **OR TI STSS** |
|  | #3 | TI prevalence OR **TI incidence** OR **TI proportion OR TI** e[pidemiology](https://www.ncbi.nlm.nih.gov/mesh/68004813) |
|  | #4 | #1 AND #2 AND #3 |

Table S2. Quality assessment results of included studies.

| Study | Q1 | Q2 | Q3 | Q4 | Q5 | Q6 | Q7 | Q8 | Q9 | Overall |
| --- | --- | --- | --- | --- | --- | --- | --- | --- | --- | --- |
| Al Barmawi et al. 2025 | Y | N | Y | Y | Y | Y | Y | Y | Y | L |
| Alshammari et al. 2024 | Y | N | Y | Y | Y | Y | Y | Y | Y | L |
| Ariapooran et al. 2022 | N | N | U | Y | Y | Y | Y | Y | U | M |
| Beck and Gable. 2012 | Y | N | U | Y | Y | Y | Y | Y | U | M |
| Beck et al. 2015 | Y | N | U | Y | Y | Y | Y | Y | U | M |
| Beck et al. 2017 | Y | N | U | Y | Y | Y | Y | Y | N | M |
| Cai et al. 2024 | Y | N | Y | Y | Y | Y | Y | Y | Y | L |
| Civljak et al. 2024 | Y | N | U | Y | Y | Y | Y | Y | U | M |
| Comparcini et al. 2025 | Y | N | U | Y | Y | Y | Y | Y | U | M |
| Dominguez-Gomez and Rutledge. 2009 | Y | N | U | Y | Y | Y | Y | Y | U | M |
| Duffy et al. 2015 | Y | N | Y | Y | Y | Y | Y | Y | U | M |
| Erkin et al. 2021 | Y | N | U | Y | Y | Y | Y | Y | Y | L |
| Frazier. 2026 | Y | N | U | Y | Y | Y | Y | Y | N | M |
| Haji Ali Begloo et al. 2025 | Y | N | Y | Y | Y | Y | Y | Y | U | M |
| He et al. 2026 | Y | N | Y | Y | Y | Y | Y | Y | U | M |
| Hu et al. 2024 | Y | N | Y | Y | Y | Y | Y | Y | N | M |
| Kellogg et al. 2018 | Y | N | Y | Y | Y | Y | Y | Y | N | M |
| Lim et al. 2026 | Y | N | U | Y | Y | Y | Y | Y | Y | L |
| Lubbad et al. 2026 | Y | N | U | Y | Y | Y | Y | Y | Y | L |
| Morrison and Joy. 2016 | Y | N | Y | Y | Y | Y | Y | Y | U | M |
| Nicholls et al. 2021 | Y | N | U | Y | Y | Y | Y | Y | Y | L |
| Ratrout and Hamdan-Mansour. 2020 | Y | N | U | Y | Y | Y | Y | Y | U | M |
| Salameh et al. 2023 | Y | N | U | Y | Y | Y | Y | Y | U | M |
| Scott et al. 2021 | Y | N | U | Y | Y | Y | Y | Y | Y | L |
| Tsouvelas et al. 2022 | Y | N | U | Y | Y | Y | Y | Y | Y | L |
| Woo and Kim. 2021 | Y | N | U | Y | Y | Y | Y | Y | Y | L |
| Yao et al. 2024 | Y | N | U | Y | Y | Y | Y | Y | Y | L |
| Yehene et al. 2024 | Y | N | U | Y | Y | Y | Y | Y | U | M |

Notes: Y: Yes, N: No, U: Unclear. L: Low risk, M: Moderate risk, H: High risk.

Q1: Was the sample frame appropriate to address the target population?

Q2: Were study participants sampled in an appropriate way?

Q3: Was the sample size adequate?

Q4: Were the study subjects and the setting described in detail?

Q5: Was the data analysis conducted with sufficient coverage of the identified sample?

Q6: Were valid methods used for the identification of the condition?

Q7: Was the condition measured in a standard, reliable way for all participants?

Q8: Was there appropriate statistical analysis?

Q9: Was the response rate adequate, and if not, was the low response rate managed appropriately?


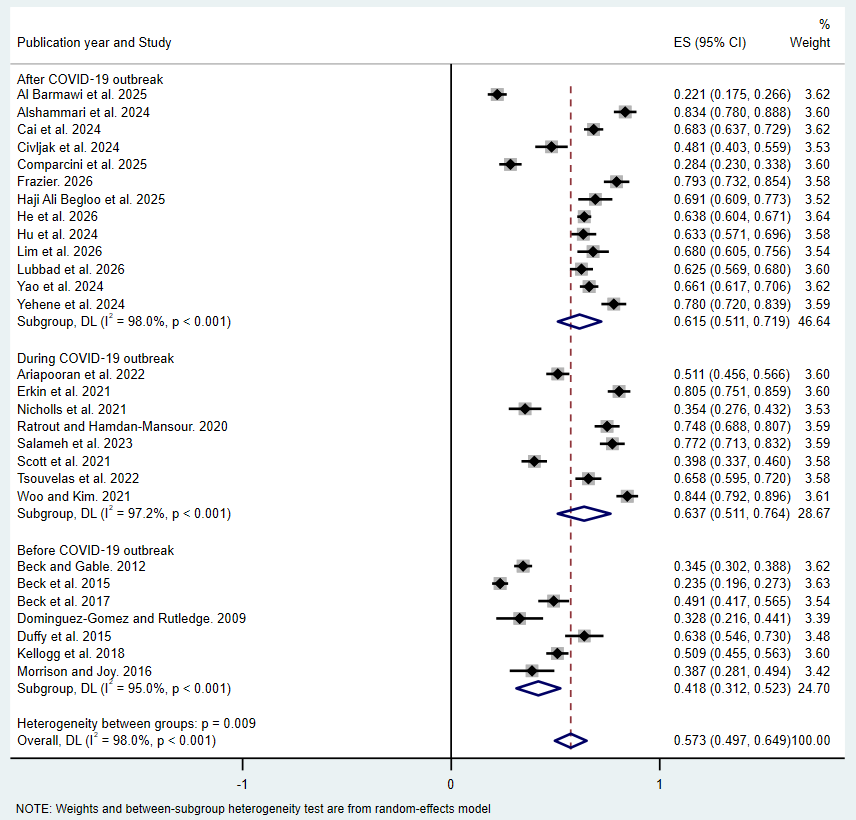


Figure S1. The pooled prevalence of secondary traumatic stress in nurses based on publication year.


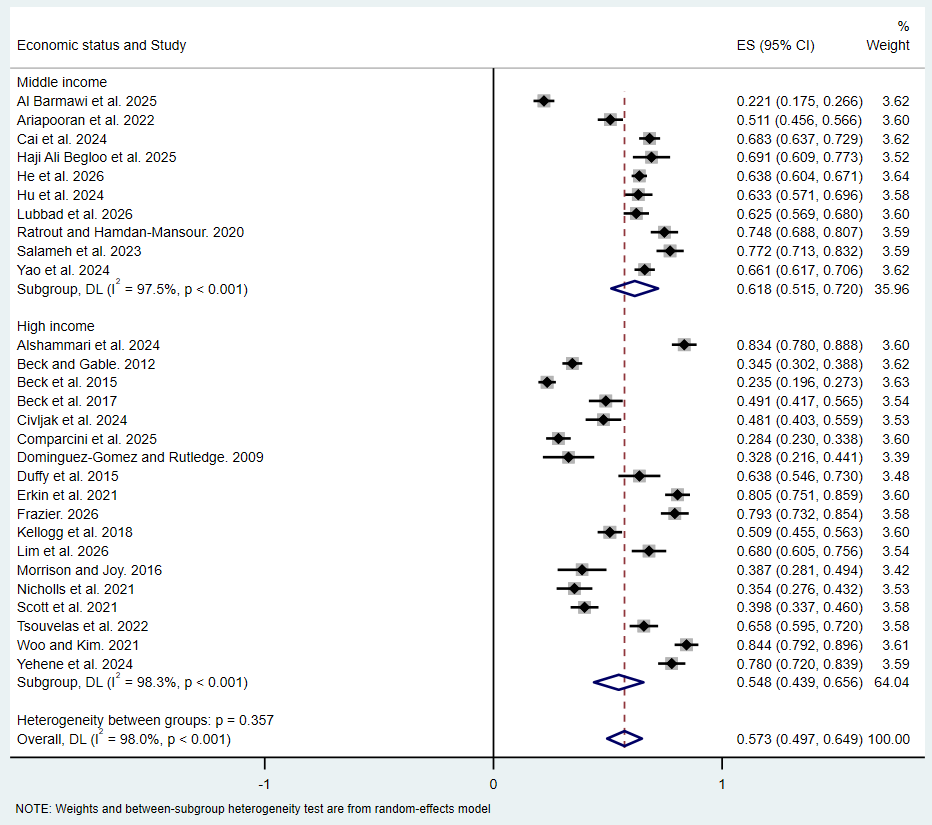


Figure S2. The pooled prevalence of secondary traumatic stress in nurses based on economic status.


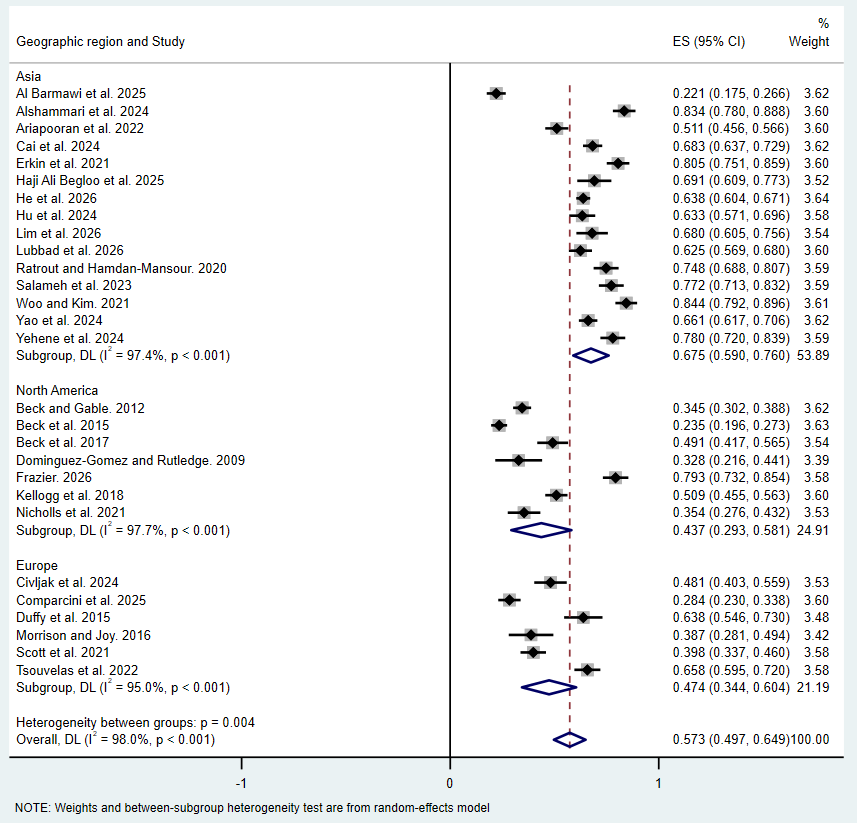


Figure S3. The pooled prevalence of secondary traumatic stress in nurses based on geographic region.


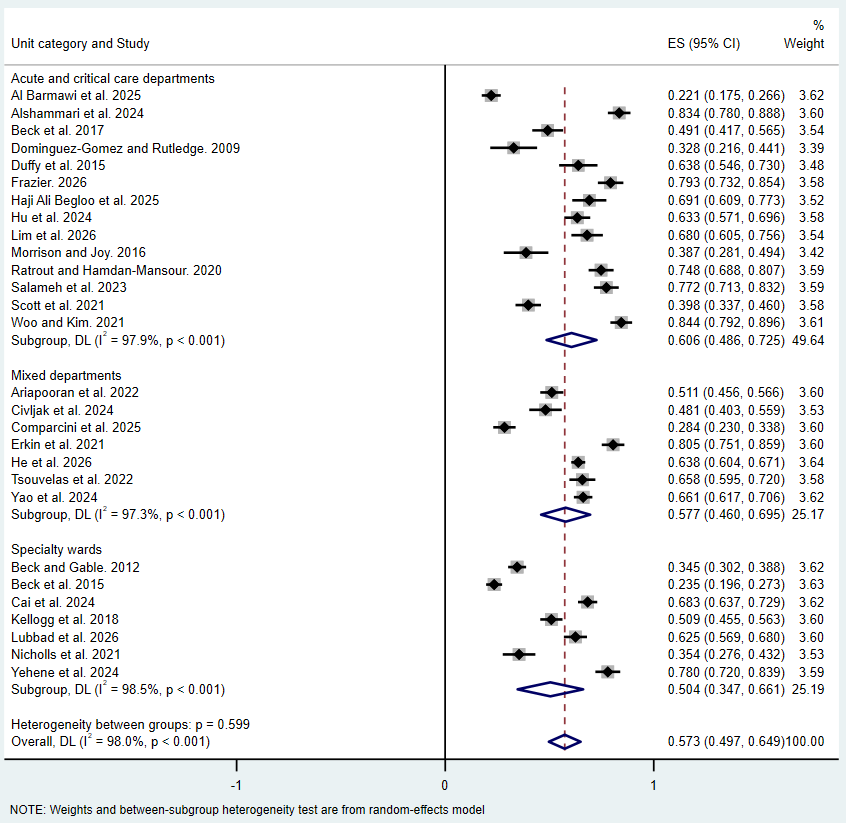
Figure S4. The pooled prevalence of secondary traumatic stress in nurses based on unit category.


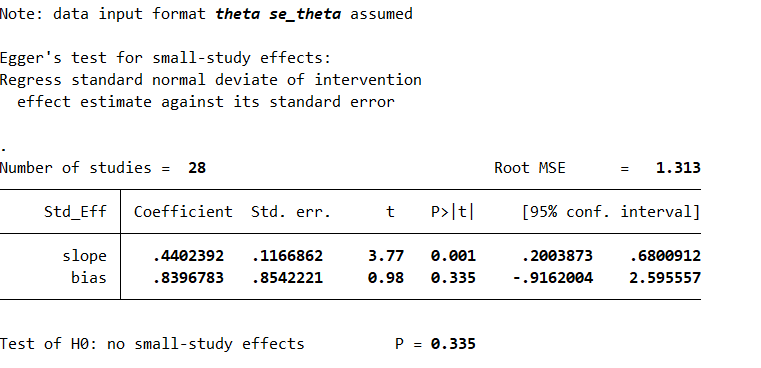


Figure S5. Egger's test results.

Table S3. The results of the sensitivity analysis.

| **Omitted Study** | **Prevalence** | **95% CI** |
| --- | --- | --- |
| Al Barmawi et al. 2025 | 0.586 | (0.514, 0.659) |
| Alshammari et al. 2024 | 0.563 | (0.487, 0.639) |
| Ariapooran et al. 2022 | 0.575 | (0.496, 0.654) |
| Beck and Gable. 2012 | 0.582 | (0.505, 0.658) |
| Beck et al. 2015 | 0.586 | (0.515, 0.657) |
| Beck et al. 2017 | 0.576 | (0.498, 0.654) |
| Cai et al. 2024 | 0.569 | (0.490, 0.648) |
| Civljak et al. 2024 | 0.576 | (0.498, 0.654) |
| Comparcini et al. 2025 | 0.584 | (0.508, 0.660) |
| Dominguez-Gomez and Rutledge. 2009 | 0.582 | (0.504, 0.659) |
| Duffy et al. 2015 | 0.571 | (0.493, 0.649) |
| Erkin et al. 2021 | 0.564 | (0.488, 0.641) |
| Frazier. 2026 | 0.565 | (0.488, 0.642) |
| Haji Ali Begloo et al. 2025 | 0.569 | (0.491, 0.647) |
| He et al. 2026 | 0.571 | (0.490, 0.651) |
| Hu et al. 2024 | 0.571 | (0.492, 0.649) |
| Kellogg et al. 2018 | 0.575 | (0.496, 0.654) |
| Lim et al. 2026 | 0.569 | (0.491, 0.647) |
| Lubbad et al. 2026 | 0.571 | (0.492, 0.650) |
| Morrison and Joy. 2016 | 0.580 | (0.502, 0.657) |
| Nicholls et al. 2021 | 0.581 | (0.504, 0.658) |
| Ratrout and Hamdan-Mansour. 2020 | 0.567 | (0.489, 0.644) |
| Salameh et al. 2023 | 0.566 | (0.488, 0.643) |
| Scott et al. 2021 | 0.579 | (0.502, 0.657) |
| Tsouvelas et al. 2022 | 0.570 | (0.491, 0.648) |
| Woo and Kim. 2021 | 0.563 | (0.487, 0.639) |
| Yao et al. 2024 | 0.570 | (0.490, 0.649) |
| Yehene et al. 2024 | 0.565 | (0.488, 0.643) |
